# Thrombospondin 1–CD47 Signalling Modulates Vascular Smooth Muscle Cell Senescence in Chronic Kidney Disease

**DOI:** 10.3390/ijms27020755

**Published:** 2026-01-12

**Authors:** Katie Trinh, Sally Coulter, Cuicui Xu, Nadia Chandra Sekar, Sohel M. Julovi, Natasha M. Rogers

**Affiliations:** 1 Kidney Injury Group, Centre for Transplant and Renal Research, Westmead Institute for Medical Research, Westmead, NSW 2145, Australia; katie.trinh@sydney.edu.au (K.T.);; 2 Renal Medicine, Blacktown Hospital, Blacktown, NSW 2148, Australia; 3 Faculty of Medicine and Health, University of Sydney, Camperdown, NSW 2050, Australia; 4 Department of Renal and Transplantation Medicine, Westmead Hospital, Westmead, NSW 2145, Australia

**Keywords:** chronic kidney disease, vascular smooth muscle cells, uraemia, senescence, thrombospondin-1, CD47

## Abstract

Chronic kidney disease (CKD) accelerates vascular dysfunction and cardiovascular disease, partly through the accumulation of the uraemic toxin indoxyl sulphate (IS). Thrombospondin-1 (TSP1) and its receptor CD47 have been implicated in vascular pathology, but their role in CKD-associated vascular remodelling is unknown. We investigated the contribution of TSP1–CD47 signalling to vascular smooth muscle cell (VSMC) dysfunction in CKD. Human aortic VSMCs (hVSMCs) were exposed to IS, TSP1, or plasma from patients with CKD. CKD was induced in wild-type (WT) and CD47-deficient (CD47KO) mice using 5/6 nephrectomy. Vascular changes were assessed by histology, immunohistochemistry, and molecular analyses. IS, TSP1, and CKD plasma increased TSP1 expression in hVSMCs, reduced proliferation, elevated β-galactosidase activity, and activated phosphorylated ERK1/2 and cytoplasmic aryl hydrocarbon receptor. These effects were attenuated by CD47 blockade. CKD plasma further enhanced IS- and TSP1-induced senescence. In vivo, 5/6 nephrectomy induced aortic wall thickening in WT but not in CD47KO mice. Aortic pERK1/2 was reduced in CD47KO mice despite persistent TSP1 upregulation. IS and TSP1 promote VSMC senescence through CD47-dependent ERK1/2 and AhR signalling. CD47 deletion protects against CKD-induced vascular remodelling, suggesting that CD47 blockade may represent a novel therapeutic strategy to mitigate vascular complications in CKD.

## 1. Introduction

Chronic kidney disease (CKD) is associated with vascular pathology, including atherosclerosis, arteriosclerosis, heterotopic medial calcification, and arterial stiffness, which contribute to the progression of cardiovascular disease (CVD) and increased mortality [1,2]. Such changes are due to long-term exposure to uraemic toxins, in addition to traditional risk factors like hypertension and hyperlipidaemia [3]. Concentrations of the highly protein-bound uraemic toxin indoxyl sulphate (IS) progressively increase with the stage of CKD and are associated with major adverse cardiovascular events (MACEs) and overall mortality [4]. IS demonstrates direct cytotoxic effects, altering vascular smooth muscle cell (VSMC) proliferation [5,6], senescence [7], migration [8], and apoptosis [9].

Thrombospondin-1 (TSP1) is a secreted matricellular glycoprotein that can interact with numerous cell-surface receptors on various cell types to modulate biological processes such as proliferation, migration, adhesion, and inflammation [10]. Plasma TSP1 expression is upregulated in CKD patients [11], and high levels are predictive of both cardiovascular and all-cause mortality in end-stage kidney disease (ESKD) [12]. Vascular expression of TSP1 is upregulated in response to injury [13], shear stress [14], and disease states, including ischemia [15] and diabetes [16]. We have previously shown that TSP1 is also increased in IS-treated VSMC [17].

The high-affinity receptor for TSP1 in VSMC is CD47 [18,19], with activation at picomolar concentrations. The importance of the TSP1-CD47 signalling axis has been identified in systemic vascular dysfunction [20], as well as pulmonary arterial hypertension [21,22]. However, its role in uraemia-induced vascular pathology has yet to be investigated. Here, we report that TSP1 and IS drive vascular proliferation and senescence via the CD47-mediated activation of AhR and pERK1/2 pathways in isolated hVSMCs. CD47 deletion protects against CKD-induced vascular remodelling, despite persistent TSP1 upregulation in mice aortae.

## 2. Results

### 2.1. Exogenous TSP1, Indoxyl Sulfate, and CKD-Patient Plasma Promote Endogenous TSP1 Expression in VSMC

**Figure 1:**
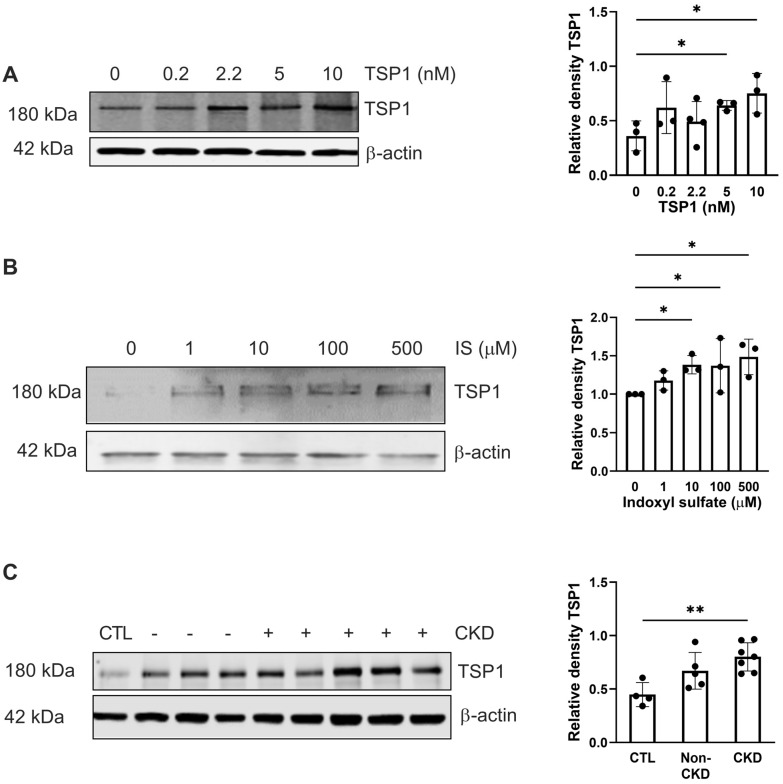
Exogenous TSP1, indoxyl sulfate, and CKD-patient plasma promote endogenous TSP1 expression in VSMC. hVSMC were treated with (**A**) TSP1 (0, 0.2, 2.2, 5, and 10 nM) (*n* = 3–4), (**B**) IS (0, 1, 10, 100, and 500 µM) (*n* = 3), or (**C**) 5% human plasma from patients with or without CKD (*n* = 3–7) for 24 h. Whole cell lysates were probed for TSP1. All data shown are mean ± SD. Representative Western blots and combined densitometry relative to β-actin are shown. * *p* < 0.05 and ** *p* < 0.01 by Kruskal–Wallis test (**A**,**B**) and one-way analysis of variance with Holm–Sidak post hoc test (**C**). Abbreviations: CKD—chronic kidney disease; CTL—control; hVSMC—human aortic vascular smooth muscle cells; IS—indoxyl sulfate; TSP1—thrombospondin-1.

The circulating levels of thrombospondin-1 (TSP1) [11] and indoxyl sulfate (IS) increase with CKD severity [23], and we have previously demonstrated that IS induces expression of TSP1 in cardiomyocytes to drive cardiac remodelling [17]. We now demonstrate in the present study that this effect is not cell-specific. hVSMCs were treated for 24 h with TSP1 (0.2 to 10 nM) (Figure 1A) and IS (1–500 µM) (Figure 1B) at concentrations consistent with those observed in CKD patients [4,23], or plasma from patients with CKD (Figure 1C). Both exogenous TSP1 and IS upregulated endogenous TSP1 expression in hVSMCs. Similarly, exposure to plasma (Figure 1C) or serum (Figure S1A) from CKD patients consistently increased endogenous TSP1 expression but not from non-CKD patients.

### 2.2. TSP1 and Indoxyl Sulfate Change VSMC Proliferation and Senescence via CD47

**Figure 2:**
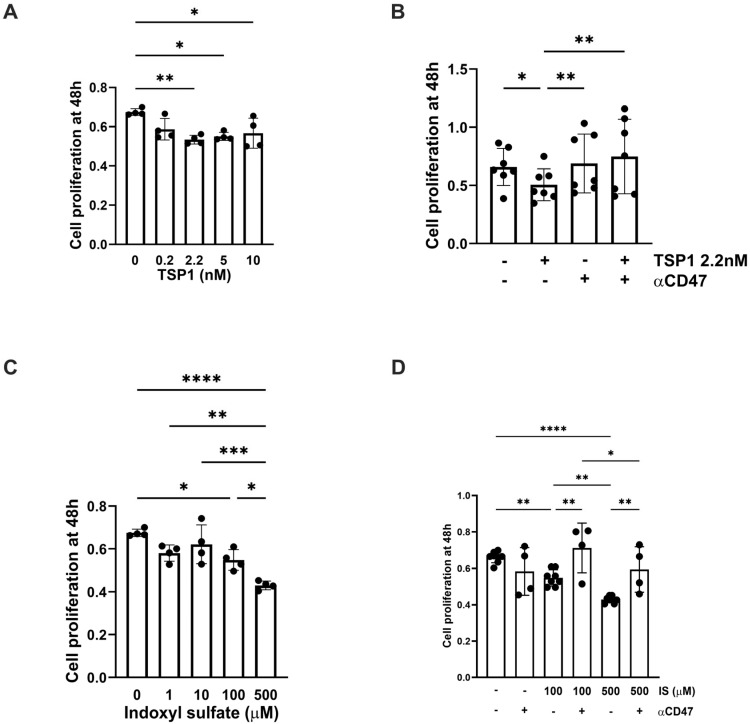
TSP1 and indoxyl sulfate limit VSMC proliferation via CD47. hVSMC cell viability was measured by assessing reduction in tetrazolium salt sodium 3′- [1- [(phenylamino)-carbonyl]-3,4-tetrazolium]-bis(4-methoxy-6-nitro)benzene-sulfonic acid hydrate (XTT) and measuring absorbance at 450 nm in cells after 48 h treatment with (**A**) TSP1 (0, 0.2, 2.2, 5, 10 nM) (*n* = 4), (**B**) TSP1 2.2 nM ± pre-treatment with anti-CD47 antibody for 30 min (*n* = 6), (**C**) IS (0, 1, 10, 100, 500 µM) (*n* = 4), or (**D**) IS (100, 500 µM) ± pre-treatment with anti-CD47 antibody for 30 min (*n* = 4–8). All data shown are mean ± SD. * *p* < 0.05, ** *p* < 0.01, *** *p* < 0.001 and **** *p* < 0.0001 by one-way analysis of variance with Holm–Sidak post-hoc test (**A**,**C**) or Kruskal–Wallis test (**B**,**D**). Abbreviations: αCD47—anti-CD47 antibody; hVSMC—human aortic vascular smooth muscle cell; IS—indoxyl sulfate; TSP1—thrombospondin-1.

TSP1 and IS are known to affect hVSMC proliferation in a time- and dose-dependent manner [24]. TSP1 at 2.2 nM (and at higher concentrations) inhibited hVMSC proliferation (Figure 2A), and this was mitigated by an anti-CD47 antibody (Figure 2B). A similar trend was seen with IS (Figure 2C), particularly at higher concentrations, which was also reversed by blocking CD47 (Figure 2D). TSP1 exerted concentration-dependent effects on VSMCs: low levels promoted proliferation, whereas higher levels suppressed it potentially by modulating cyclic nucleotide levels (cAMP/cGMP) and interfering with nitric oxide (NO) signalling, consistent with our culture conditions [25,26,27]. These opposing actions likely reflect signals from distinct TSP1 domains and receptor pathways [26], and exogenous TSP1 at 2.2 nM was reported to engage CD47 in vitro [28], which is consistent with Figure 2B,D.

**Figure 3:**
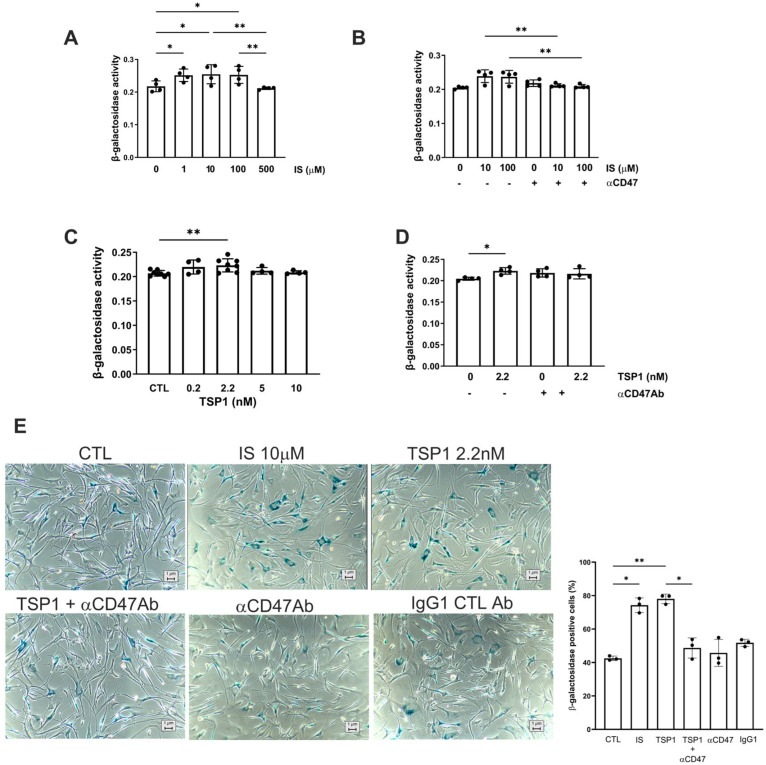
TSP1 and indoxyl sulfate change VSMC senescence via CD47. hVSMC senescence-associated β-galactosidase activity after 48 h treatment with (**A**) IS (0, 1, 10, 100, 500 µM) (*n* = 4), (**B**) IS (10, 100 µM) ± pre-treatment with anti-CD47 antibody for 30 min, (**C**) TSP1 (0, 0.2, 2.2, 5, 10 nM) (*n* = 4–7), or (**D**) TSP1 2.2 nM ± pre-treatment with anti-CD47 antibody for 30 min (*n* = 4). (**E**) Senescence-associated β-galactosidase staining of hVSMC following incubation with basal media (CTL), IS 10 µM (±pre-treatment with anti-CD47 antibody for 30 min), TSP1 2.2 nM (±pre-treatment with anti-CD47 antibody for 30 min), and IgG isotype control antibody (*n* = 3). All data shown are mean ± SD. * *p* < 0.05 and ** *p* < 0.01 by one-way analysis of variance with Holm–Sidak test (**A**–**C**) and Kruskal–Wallis test (**D**,**E**). Abbreviations: αCD47Ab—anti-CD47 antibody; CTL—control; hVSMC—human aortic vascular smooth muscle cell; IS—indoxyl sulfate; TSP1—thrombospondin-1.

Cellular senescence contributes to vascular pathology through oxidative stress, inflammation, and mitochondrial dysfunction, which is attributed, in part, to the retention of uremic toxins [29]. IS appeared to have a biphasic effect on hVSMC senescence (Figure 3A), although β-galactosidase activity at lower concentrations was reduced by pre-treatment with αCD47Ab (Figure 3B,E). TSP1 (at 2.2 nM) also increased senescence in hVSMCs (Figure 3C), which was reduced by blocking CD47 signalling (Figure 3D,E).

### 2.3. TSP1 Activates AhR in VSMC via CD47

**Figure 4:**
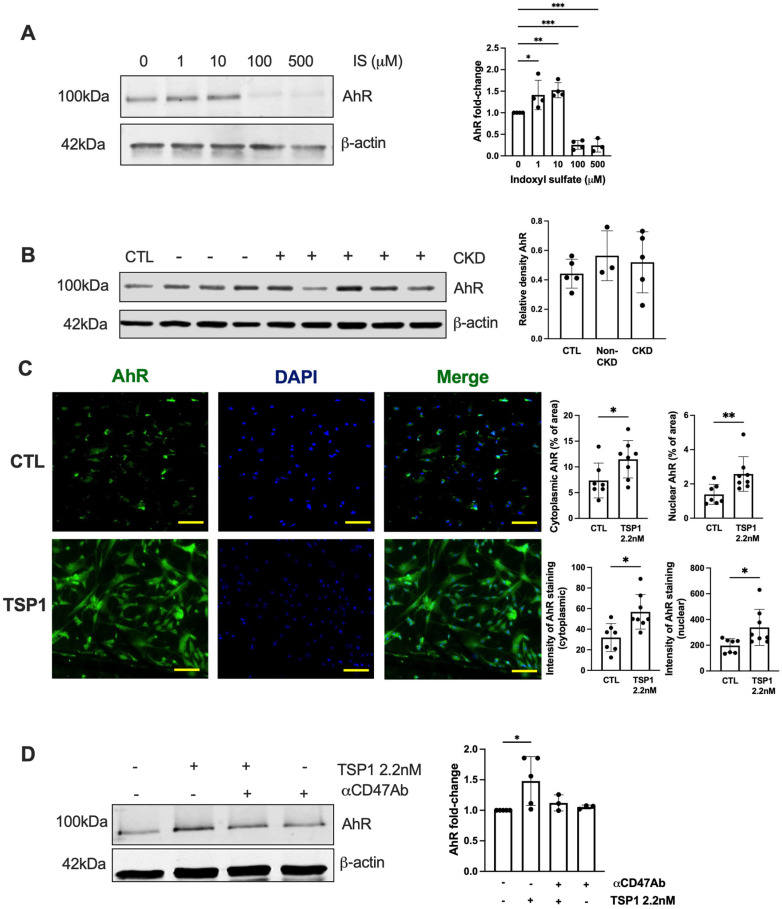
TSP1 activates AhR in VSMC via CD47. hVSMC were treated with (**A**) IS (0, 1, 10, 100, 500 µM) (*n* = 3–4) or (**B**) 5% human plasma from patients with or without CKD (*n* = 3–5) for 24 h. Whole cell lysates were probed for AhR. Representative Western blots and combined densitometry relative to β-actin or vinculin are shown. (**C**) hVSMC treated with TSP1 2.2 nM were stained for AhR (green) and 4′,6-diamidino-2-phenylindole (blue) (Magnification = 40×, Scale bar = 80 pixels). Percentage and intensity of cytoplasmic and nuclear staining were measured (*n* = 7–8). All data shown are mean ± SD. * *p* < 0.05, ** *p* < 0.01, and *** *p* < 0.001 by one-way analysis of variance with Holm–Sidak’s post hoc test (**A**,**B**,**D**) and unpaired Student’s *t*-test (**C**). Abbreviations: AhR—aryl hydrocarbon receptor; CKD—chronic kidney disease; hVSMC—human aortic vascular smooth muscle cell; IS—indoxyl sulfate; TSP1—thrombospondin-1.

IS activates the aryl hydrocarbon receptor (AhR) [30,31], driving transcriptional programmes that exacerbate systemic oxidative stress and inflammation, which are all elevated in CKD. We reported previously that AhR and TSP1 are associated with cardiorenal syndrome [17], and CKD through IS and TSP1 induces AhR myocardial expression. AhR expression in hVSMCs in response to exogenous IS was biphasic (Figure 4A). Plasma from either healthy controls or CKD patients was unable to stimulate AhR expression in hVSMCs (Figure 4B). TSP1 activated both cytoplasmic- and nuclear-AhR expression in hVSMCs (Figure 4C), and pre-treatment with αCD47Ab reduced TSP1-induced cytoplasmic-AhR expression (Figure 4D).

### 2.4. Establishing Equivalent CKD in WT and CD47KO Mice

**Figure 5:**
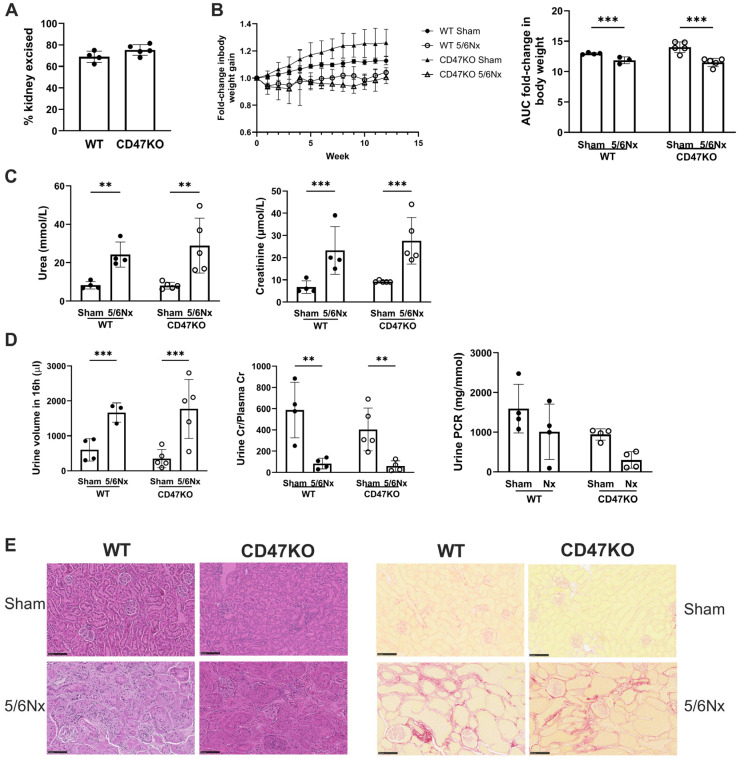
Establishing equivalent CKD in WT and CD47KO mice. WT and CD47KO mice were subjected to 5/6Nx or sham surgery. (**A**) Percentage of kidney excised following 5/6Nx (*n* = 4–5). (**B**) Fold-change in body weight over 12 weeks (*n* = 3–5). (**C**) Serum urea and creatinine at 12 weeks post-surgery (*n* = 4–5). (**D**) Urine volume, urine-to-plasma creatinine ratio, and urine protein-to-creatinine ratio at 12 weeks (*n* = 3–5). (**E**) Representative haematoxylin and eosin-stained sections of formalin-fixed paraffin-embedded kidney excised at 12 weeks following 5/6Nx or sham surgery (bar = 100 µm). All data shown are mean ± SD. ** *p* < 0.01, and *** *p* < 0.001 by unpaired Student’s *t*-test (**A**) or 2-way analysis of variance with Holm–Sidak post hoc test (**B**–**D**). Abbreviations: 5/6Nx—5/6-nephrectomy; AUC—area under the curve; CD47KO—CD47-knockout; Cr—creatinine; PCR—protein-to-creatinine ratio; WT—wild-type.

CD47KO mice are known to be protected from exogenous stressors, leading to reduced susceptibility to ischemia–reperfusion injury [32]; therefore, an acute-on-chronic insult model would be difficult to achieve parity of injury compared to wild-type (WT) mice. We established a 5/6Nx model of CKD in C57BL/6 mice [17,33], which was successfully recapitulated in CD47KO mice. An equivalent proportion of kidney mass was excised in WT and CD47KO 5/6Nx groups (Figure 5A). All 5/6Nx mice (regardless of genotype) demonstrated reduced body weight gain over 12 weeks compared to sham-operated controls (Figure 5B). The development of CKD was confirmed by significantly increased plasma urea and creatinine in 5/6Nx groups, which were equivalent between WT and CD47KO mice (Figure 5C). Polyuria and reduced urine-to-plasma creatinine ratios were also observed in 5/6Nx groups (Figure 5D), consistent with our previous reports [17,33]. Urine protein-to-creatinine ratios were unchanged (Figure 5D). Kidney histology at 12 weeks following 5/6Nx demonstrated equivalent tubular atrophy, interstitial fibrosis and perivascular fibrosis in both genotypes (Figure 5E).

### 2.5. Development of CRS Upregulates Aortic TSP1 Expression

**Figure 6:**
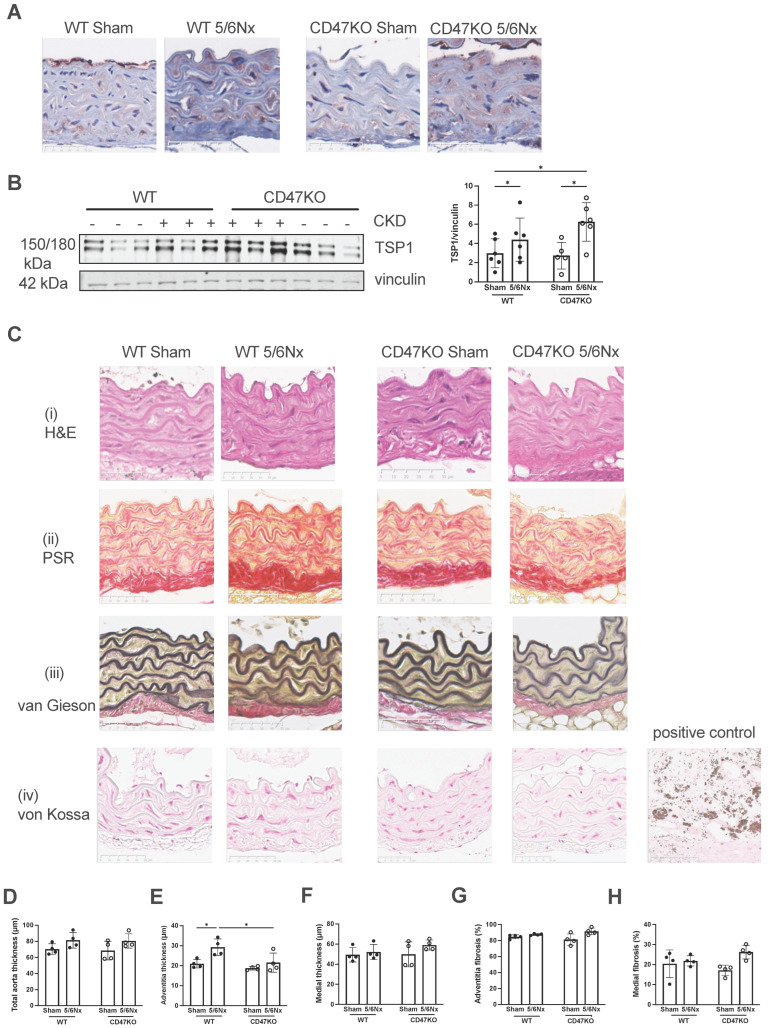
Development of CRS in mice upregulates aortic TSP1 expression. (**A**) Representative images of immunohistochemical staining for TSP1 in formalin-fixed paraffin-embedded mouse thoracic aorta. Scale bar = 50 µm. (**B**) Mouse thoracic aorta homogenates obtained at 12 weeks following 5/6Nx or sham surgery were analysed by Western blotting for expression of TSP1 (*n* = 5–6 per group). Representative Western blots and combined densitometry relative to vinculin are shown. (**C**) Representative images of formalin-fixed paraffin-embedded mouse thoracic aorta 12 weeks following 5/6Nx or sham surgery with (**i**) haematoxylin-eosin, (**ii**) picrosirius red, (**iii**) Verhoeff–van Gieson and (**iv**) von Kossa staining. Calcified human carotid atheromatous plaque was used as a positive control. Scale bar = 50 µm. Total aorta thickness (**D**), adventitia thickness (**E**), and media thickness (**F**) were measured at 5 randomly selected areas per mouse and averaged (*n* = 4/group). Collagen deposition in the aortic adventitia (**G**) and media (**H**) was quantified from 5 randomly selected areas per mouse and averaged (*n* = 4/group) in picrosirius-red-stained sections. All data shown are mean ± SD. * *p* < 0.05 by 2-way analysis of variance with Holm–Sidak post hoc test (**B**,**D**–**H**). Abbreviations: 5/6Nx—5/6 nephrectomy; TSP1—thrombospondin-1.

We have previously demonstrated that our pre-clinical CRS model elevates plasma IS [17], consistent with human CKD, which is strongly linked with cardiovascular pathology. We then investigated morphological changes in the thoracic aorta to determine the impact of uremia and disrupted CD47 signalling. As shown previously [17], the 5/6Nx model does not significantly change blood pressure or heart rate (Figure S2), excluding the contribution of increased afterload to vascular changes. CKD, regardless of genotype, significantly enhanced TSP1 expression (Figure 6A,B), with a trend towards increased total aorta thickness (Figure 6C, panel (i), Figure 6D). Adventitial thickness was significantly increased only in WT 5/6Nx mice (Figure 6E), and there were no differences in medial thickness (Figure 6F). Picrosirius red-stained sections (Figure 6C, panel (ii)) demonstrated no significance differences in adventitial (Figure 6G) and medial fibrosis (Figure 6H) in any experimental groups between genotypes. There was no significant difference in elastin or intimal thickening (Figure 6C, panel (iii)), and no vascular calcification was detected across all experimental groups (Figure 6C, panel (iv)).

### 2.6. MAPK ERK Signalling Is Activated by TSP1 and IS via CD47

**Figure 7:**
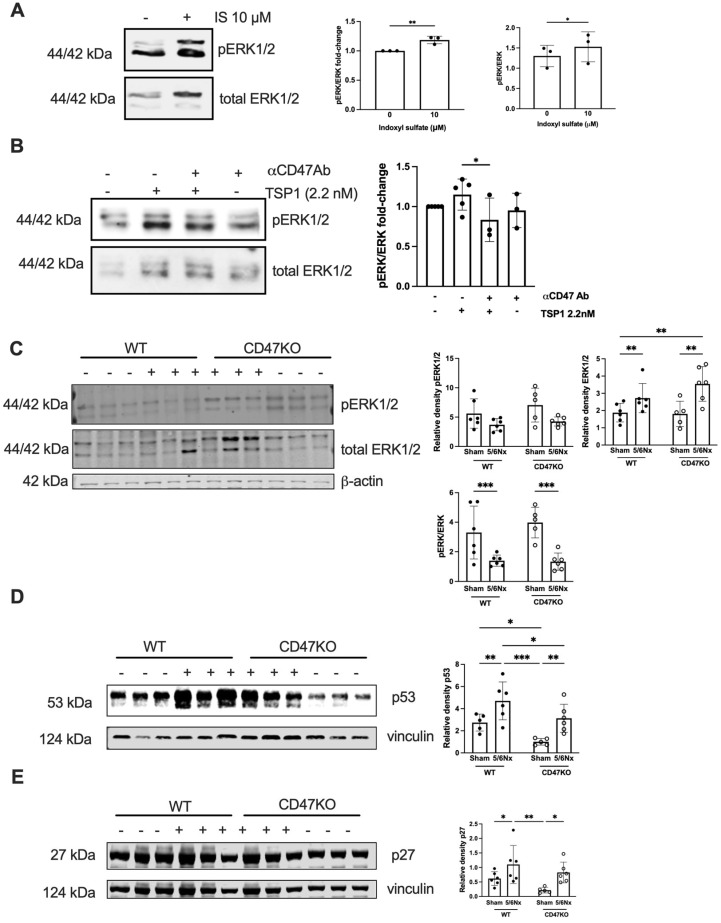
MAPK ERK signalling is activated by TSP1 and IS via CD47. hVSMC were treated for 24 h with (**A**) IS (0, 1, 10, 100, and 500 µM) (*n* = 3) or (**B**) TSP1 2.2 nM ± pre-treatment with anti-CD47 antibody for 30 min (*n* = 3–5). Whole cell lysates were probed for pERK. Mouse thoracic aorta homogenates obtained at 12 weeks following 5/6Nx or sham surgery were analysed by Western blotting for expression of pERK1/2 and total ERK1/2 (**C**), p53 (**D**), and p27 (**E**) (*n* = 5–6/group). All data shown are mean ± SD. Representative Western blots and combined densitometry relative to total ERK1/2, β-actin, or vinculin are shown. * *p* < 0.05, ** *p* < 0.01, and *** *p* < 0.001 by unpaired Student’s *t*-test (**A**), Kruskal–Wallis test (**B**), and 2-way analysis of variance with Holm–Sidak post hoc test (**C**–**E**). Abbreviations: αCD47Ab—anti-CD47 antibody; CD47KO—CD47-knockout; hVSMC—human aortic vascular smooth muscle cells; IS—indoxyl sulfate; pERK—phosphorylated extracellular regulated kinase; TSP1—thrombospondin-1; WT—wild-type.

AhR regulates TSP1 gene promoter activity [34], and mitogen-activated protein kinase (MAPK) activation has also been shown to facilitate AhR activity [35]. We have previously shown that TSP1 induces AhR and MAPK expression, suggesting multi-directional potentiation of signalling [17]. The MAPK extracellular signal-regulated kinase (ERK) plays an important role in VSMC proliferation [36,37,38]. Treatment of hVSMCs with IS upregulated phoshorylated extracellular regulated kinase (pERK) expression (Figure 7A). TSP1 also upregulated pERK expression, which was mitigated by anti-CD47 antibody (Figure 7B). The in vitro effect was not recapitulated in vivo, as expression of pERK was reduced in thoracic aortae of both genotypes following 5/6Nx (Figure 7C), although total ERK was generally increased.

### 2.7. CRS Mice Demonstrate Increased Vascular Senescence via CD47

In CKD, oxidative stress, dysregulated calcium and phosphate levels, and uremic toxins (including IS) promote DNA damage and cellular dysfunction that culminate in premature senescence. The aortae from WT 5/6Nx mice also showed amplified expression of senescence markers p53 and p27, which was significantly lower in CD47KO 5/6Nx mice (Figure 7D,E).

## 3. Discussion

CKD is strongly associated with vascular dysfunction and premature vascular ageing [39,40], but the molecular mediators remain incompletely defined. In this study, we have identified TSP1 as a key regulator of uremic vascular changes. We demonstrate that both TSP1 and the uremic toxin IS consistently reduce VSMC proliferation and induce senescence. TSP1 expression in VSMCs was also induced by IS, as well as by plasma and serum from patients with CKD, putatively containing both IS and TSP1. Our mechanistic studies revealed that both IS and TSP1 increased AhR expression and ERK1/2 phosphorylation in VMSC. The biphasic effect of IS—possibly due to altered nuclear/cytoplasmic trafficking of AhR—remains to be determined [41]. The blockade of CD47, a high-affinity TSP1 receptor, attenuated senescence and ERK activation, supporting the notion of CD47 as a critical mediator. These findings highlight TSP1 as a driver of uremic signalling and a key therapeutic target.

Although ERK is classically associated with mitogenic signalling [42], sustained activation can instead trigger cell-cycle arrest and/or senescence [43]. The amplitude and duration of ERK signalling are key determinants: moderate activation promotes cell-cycle entry, whereas strong or prolonged signalling induces p53, p21, and p27 expression, as well as growth arrest in different cell types [43,44]. Our finding that TSP1- and IS-induced ERK activation reduced proliferation and increased senescence in VMSCs is in keeping with these findings.

Our 5/6Nx model yielded divergent vascular responses. In thoracic aortae, CKD increased total ERK1/2 expression but decreased activation (phospho-ERK) in both WT and CD47KO mice. This may reflect nuclear translocation of ERK1/2 [45,46] or the influence of additional vascular wall intercellular signalling, including endothelial cells [47,48,49]. We observed a biphasic response in cytoplasmic-AhR expression in VSMC, but no corresponding activation of ERK1/2 expression. The effect of IS on ERK1/2 activation is variable and context-dependent, with some studies showing it enhances or has no effect and others demonstrating inhibition of ERK1/2 phosphorylation, depending on the cell type and cell culture conditions, requiring additional pro-inflammatory cytokines [50] or variable IS concentrations [51]. IS has been shown to activate ERK1/2 in renal proximal tubular cells [52] and VSMCs [53], while inhibiting it at higher concentrations in osteoblast-like cells [54]. These discrepancies highlight the complexity of uremic signalling in the intact vascular wall, where gradients of toxins (IS), matrix proteins (TSP1), and receptor expression influence responses.

### 3.1. Study Limitations

Several limitations should be acknowledged. First, our in vitro experiments were performed in isolated hVSMCs, which may not fully capture the multicellular interactions that occur within the vessel wall. Endothelial cells, fibroblasts, and infiltrating immune cells are likely to influence TSP1 signalling and senescence. Second, the concentrations of IS and TSP1 used in vitro may not precisely reflect the in vivo milieu, where toxin accumulation and spatial gradients are dynamic and patient-dependent. Third, we used global CD47-deficient mice, and the cell-type-specific roles of CD47 remain to be defined. Fourth, ERK1/2 activation was measured in bulk vascular tissue, which may obscure cell-specific differences in signalling. Beyond CD47, TSP1 drives VSMC remodelling through CD36-dependent, cyclin-A-mediated proliferation, contributing to neointimal hyperplasia. TSP1–integrin signalling also regulates adhesion, migration, and phenotypic switching, though its context-specific relevance remains unclear in our study [55,56,57].

Finally, the demographic characteristics of patients and their serum concentrations of IS and TSP1 are unknown, which may account for the variability in cytoplasmic AhR expression induced by serum in hVSMCs.

### 3.2. Clinical Perspectives

Despite these limitations, our findings provide novel insights with direct clinical implications. By linking IS to increased TSP1 expression and identifying a CD47–AhR–ERK signalling pathway that drives VSMC senescence, we propose a potential mechanism underlying vascular remodelling and premature vascular ageing in CKD. Consistent with this, we observed the overexpression of p27 and p53 in CKD aortae (graphical abstract). Senescent VSMCs lose contractile function, secrete extracellular matrix, and promote fibrosis, collectively contributing to heightened cardiovascular risk.

Currently, therapies targeting vascular ageing in CKD are limited. Our results suggest that blocking TSP1 or CD47-dependent signalling could mitigate vascular senescence and remodelling. Given the availability of experimental CD47-blocking antibodies and the development of small-molecule inhibitors, translational opportunities are emerging. Furthermore, circulating TSP1 may complement existing markers of uremic toxicity. Thus, targeting TSP1-driven signalling may represent a novel strategy to reduce the burden of cardiovascular disease in CKD patients.

## 4. Materials and Methods

### 4.1. Animals

CD47KO (B6.129S7-Cd47tm1Fpl/J) mice on a C57BL/6J background (back-crossed for 15 generations) were purchased from the Jackson Laboratory, then bred and housed at Australian Bioresources (ABR, Sydney, Australia). Age-matched (6–8 weeks old) male littermate control (wild-type, WT) mice purchased from ABR and homozygous CD47KO mice were transferred to the Westmead Institute for Medical Research and allowed to acclimatise for 2 weeks prior to study commencement. Mice were housed in the biological facility under a 12 h light/dark cycle with free access to standard chow and water. Animal studies were performed under approved protocols (Western Sydney Local Health District #4304, #4281 and University of Sydney #1594, #2022/2028) and in accordance with the Australian code for the care and use of animals for scientific purposes (National Health and Medical Research Council).

### 4.2. CKD Model

Age-matched (8–10 weeks old) mice were randomly assigned to sham and 5/6 nephrectomy groups (5/6Nx). The 5/6Nx model was performed as previously described [17,33]. Briefly, mice were anaesthetised with isoflurane and oxygen. In the 5/6Nx group, a left 2/3 nephrectomy was firstly performed by cutting the upper and lower poles of the left kidney with iris scissors, followed by a total right nephrectomy 7 days later. The sham groups were similarly anaesthetised and kidneys exposed but not resected. All mice were assessed weekly for body weight and blood pressure using tail-cuff plethysmography (CODA machine, Kent Scientific Corporation, Torrington, CT, USA). Metabolic caging was performed at week 10, and mice were euthanised at week 12. Organs, plasma, and urine were collected and stored at −80 °C. The percentage of the kidney excised was calculated by the following formula: % of removed kidney = weight of excised kidney (left excised kidney + right kidney) × 100/total weight (left excised kidney + left remnant kidney + right kidney).

### 4.3. Measurement of Renal Function

Plasma urea and creatinine, as well as urinary protein and creatinine, were measured using the Siemens Atellica System (Institute of Clinical Pathology and Medical Research, Westmead Hospital, Westmead, NSW, Australia).

### 4.4. Aorta and Kidney Histopathology

Formalin-fixed kidneys and thoracic aortae were embedded in paraffin and cut into 4 μm sections for histology. Slides were deparaffinized in xylene and rehydrated in ethanol prior to staining. Following staining, slides were rehydrated in ethanol, cleared in xylene, cover slipped with mounting media and left to dry overnight prior to imaging.

For haematoxylin and eosin staining, slides were stained with Mayer’s haematoxylin for 3 min, Scott’s solution for 1 min, and counterstained with eosin for 2 min.

For picrosirius-red staining (for collagen), slides were incubated in picrosirius-red staining solution for 1 h and washed in acidified water.

For Verhoeff–van Gieson staining (for elastic fibres), slides were incubated with Verhoeff staining solution for 25 min, rinsed in water, then differentiated with 2% aqueous ferric chloride and counterstained with van Gieson solution (POCD Scientific, North Rocks, NSW, Australia).

For Von Kossa staining (for calcification), slides were incubated with 1% silver nitrate under UV light for 30 min, followed by 5% sodium thiosulfate for 5 min prior to counterstaining with nuclear fast red solution for 5 min.

### 4.5. Immunohistochemistry

Four (4) μm sections of formalin-fixed paraffin-embedded mouse thoracic aorta were incubated with rabbit anti-TSP1 antibody (15 μg/mL, ab85762, Abcam, Cambridge, UK). EnVision+ System-HRP Labelled Polymer anti-Rabbit secondary antibody (K4003, Dako, Santa Clara, CA, USA) and ImmPACT NovaRED^®^ Substrate Kit Peroxidase (HRP) (SK-4805, Dako) were used for immunodetection. Slides were counterstained with Mayer’s haematoxylin (POCD Scientific) and Scott’s Blue solution (POCD Scientific).

### 4.6. Immunofluorescence

Cells were grown on glass-bottom dishes (MatTek, Ashland, MA, USA), and TSP1 (2.2 nM) was added for 24 h [11]. Permeabilisation was performed with PBS/10% BSA/0.1% Triton-X100 for 10 min at room temperature, followed by blocking with 1% goat serum (Sigma-Aldrich, St. Louis, MO, USA) for 30 min. Cells were incubated in primary antibody (1:100 dilution) in blocking buffer in a humidified chamber at 4 °C overnight. Cells were washed, then incubated with AF488 secondary antibody (1:400 dilution, Invitrogen, Waltham, MA, USA) for 1 h. Nuclei were stained with DAPI. Cells were coverslipped with Gelvatol mounting media. Images were captured with an Olympus Fluoview 1000 confocal microscope (Olympus, Tokyo, Japan), and results were calculated as the percentage of area stained using ImageJ (Version 2.14.0/1.54f, National Institutes for Health, Bethesda, MD, USA).

### 4.7. Slide Imaging and Analysis

Slides were scanned under brightfield conditions using a Nanozoomer Slide scanner (Hamamatsu Photonics, Shizuoka, Japan) and viewed on NDP.view2 (Hamamatsu, Japan). Image analysis was conducted using ImageJ. Medial, adventitial, and total wall thickness were measured at 5 randomly selected locations, and the average was calculated. The percentage of medial and adventitial fibrosis was assessed by taking the average of picrosirius-red staining of collagen in 5 randomly selected high-power fields at 80× magnification per specimen, as per previously published methods [58].

### 4.8. Human Plasma Collection

Human plasma and serum collection and study were performed under protocols approved by the Human Research Ethics Committee of Western Sydney Local Health District [2019/PID02481 (LNR/16/WMEAD/399), with approval dates from 11 November 2016 to 30 December 2022]. Patients without intercurrent illness or acute kidney injury were recruited from outpatient clinics and provided written consent. Plasma samples were obtained from patients with estimated glomerular filtration rate (eGFR) > 90 mL/min/1.73 m^2^ (control) and those with stage 5 CKD (eGFR < 15 mL/min). Blood was collected in EDTA tubes using a 23-gauge needle and without a tourniquet. Tubes were placed immediately on ice, then centrifuged at 2500 rpm for 15 min at 4 °C without brake to generate platelet-poor plasma, which was stored at −80 °C until use.

### 4.9. Cell Culture

Human aortic smooth muscle cells (hVSMCs) (CC-2571, Clonetics, Lonza, Basel, Switzerland) were cultured in SmBM™ basal medium (CC-3181, Lonza) supplemented with SmGM™-2 SingleQuots growth supplements (CC-4149, Lonza). Cells were subcultured according to manufacturer’s instructions. Cells were used at passages 3–6 when at ~70% confluency in 6-well plates. Serum-starved cells were treated with TSP1 (isolated from human platelets) (0.2–10 nM, Athens Research and Technology, Athens, GA, USA), IS (1–500 μM, I3875, Sigma-Aldrich), or 5% human non-CKD and CKD plasma or serum for 24 h. In some experiments, cells were pre-treated with anti-CD47 antibody (B6H12) (#14-0479-82, Thermo Fisher Scientific, Waltham, MA, USA) for 30 min prior to treatment with TSP1.

### 4.10. Cell Proliferation and Senescence Assays

Cells were seeded into a 96-well microplate (10^4^ cells/well). Serum-starved cells were treated with human plasma, TSP1, or IS for 24 or 48 h, as indicated. In some experiments, cells were pre-treated with anti-CD47 antibody for 30 min. Cell proliferation and senescence were measured using XTT Cell Viability Kit (#9095, Cell Signalling Technology, Danvers, MA, USA) and Mammalian β-Galactosidase Assay Kit (75707, Thermo Fisher Scientific), respectively. Absorbance was measured using SpectraMax iD5 Plate Reader (Molecular Devices, San Jose, CA, USA) at 450 nm for cell proliferation and 405 nm for cell senescence [17]. In additional experiments, cells were seeded onto 4-well glass slides (10^4^ cells/slide) and stained using a senescence β-galactosidase staining kit (#9680, Cell Signalling Technology) as per manufacturer’s instructions. Mouse IgG1 kappa isotype control (17-4714-42, eBioscience, San Diego, CA, USA) was used. Cells were examined under a bright-field microscope (Olympus CKX53, Evidence Scientific, Tokyo, Japan), and five random images were captured per slide for β-galactosidase-positive (blue) cells.

### 4.11. Western Blotting

Mouse thoracic aortic tissue or human aortic smooth muscle cells were homogenised in RIPA buffer (#9806, Cell Signalling Technology) containing protease inhibitor cocktail (cOmplete^TM^ ULTRA Tablets 5892970001, Roche, North Ryde, Australia) and phosphatase inhibitor cocktail (PhosSTOP^TM^ 4906845001, Roche). Protein concentration was measured using DC protein assay (Biorad, Hercules, CA, USA), then resolved by sodium dodecyl sulfate polyacrylamide gel electrophoresis and transferred onto nitrocellulose membranes by Trans-Blot Turbo Transfer System (Biorad). Membranes were blocked with Intercept^®^ Blocking Buffer (927-60001, LICORbio, Lincoln, NE, USA) and probed at 4 °C overnight with the following primary antibodies: phospho-p44/42 MAPK (pERK1/2, #4370), p44/42 MAPK (ERK1/2, #9107), β-actin (#4970), vinculin (#13901), AhR (#83200), tumour protein p53 (p53) (#2524), and p27^Kip1^ (#3698) from Cell Signalling Technology and TSP1 (ab85762) from Abcam. IRDye 800CW and 680RD secondary antibodies (LICORbio) were used, and protein was visualised using Odyssey CLx Imaging System (LICORbio). Protein band intensity was measured using ImageJ and expressed relative to loading controls, vinculin or β-actin.

### 4.12. Statistical Analysis

Statistical analyses were performed using GraphPad Prism Version 10.2.3 (GraphPad Software Inc.). Data are presented as mean ± SD. Unpaired *t*-test (2 comparator groups) or one-way or two-way analysis of variance with Holm–Sidak post hoc test and Kruskal–Wallis test (>2 comparator groups) was used. A *p*-value less than 0.05 was considered statistically significant.

## Data Availability

The original contributions presented in this study are included in the article/Supplementary Materials. Further inquiries can be directed to the corresponding authors.

## Supplementary Materials

The supporting information can be downloaded at: https://www.mdpi.com/article/10.3390/ijms27020755/s1.

## Abbreviations

The following abbreviations are used in this manuscript:

αCD47Ab	Anti-CD47 antibody
hVSMC	Human aortic vascular smooth muscle cell
IS	Indoxyl sulfate
TSP1	Thrombospondin-1
CKD	Chronic kidney disease
AhR	Aryl hydrocarbon receptor
5/6Nx	5/6-nephrectomy
Cr	Creatinine
PCR	Protein-to-creatinine ratio
WT	Wild-type
pERK	Phosphorylated extracellular-regulated kinase
