# Supplementary material for: Thrombospondin 1–CD47 Signalling Modulates Vascular Smooth Muscle Cell Senescence in Chronic Kidney Disease
Source: Int J Mol Sci. 2026 Jan 12;27(2):755. doi: 10.3390/ijms27020755 (PMC12840690; doi:10.3390/ijms27020755)
Supplement: Supplementary file 1 [file ijms-27-00755-s001.zip › ijms-3915237-supplementary.pdf]

Supp. Figure S1.

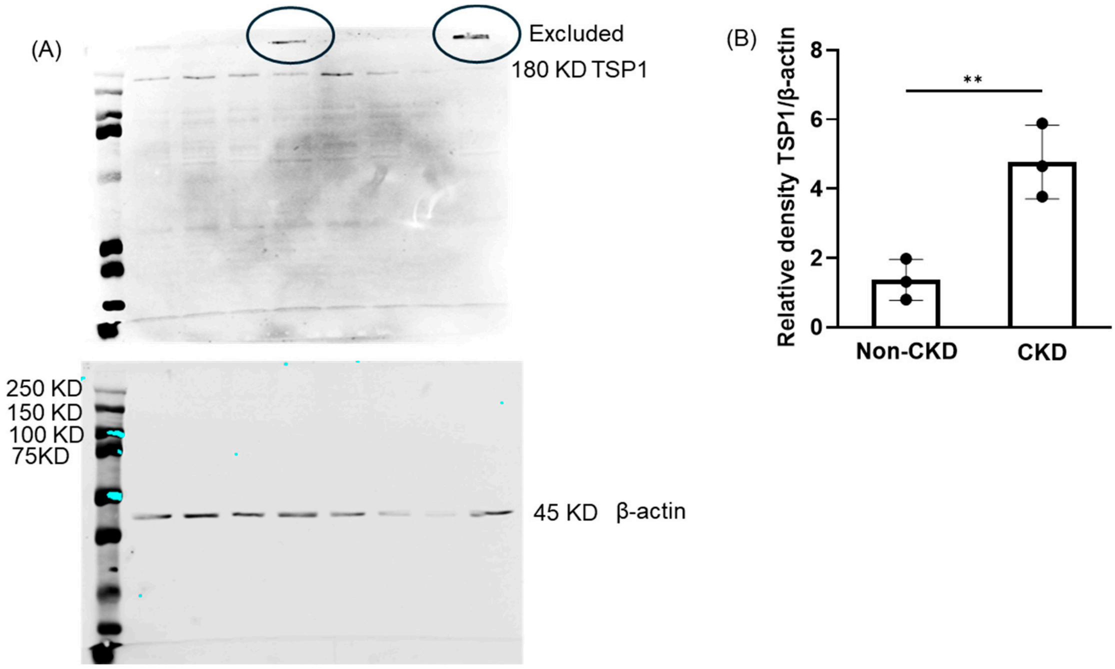

Figure S1. Expression of TSP1 by non-CKD and CKD serum in hVSMC. Human vascular smooth muscle cells were (hVSMCs) were treated with 5% human serum from patients with or without CKD (n=4) for 24h. Whole cell lysates were probed for TSP1. All data shown are mean ± SD. (A) Representative Western blots and (B) combined densitometry relative to β-actin are shown. *P<0.05 and **P<0.01 by unpaired students t-test.

Supp. Figure S2.

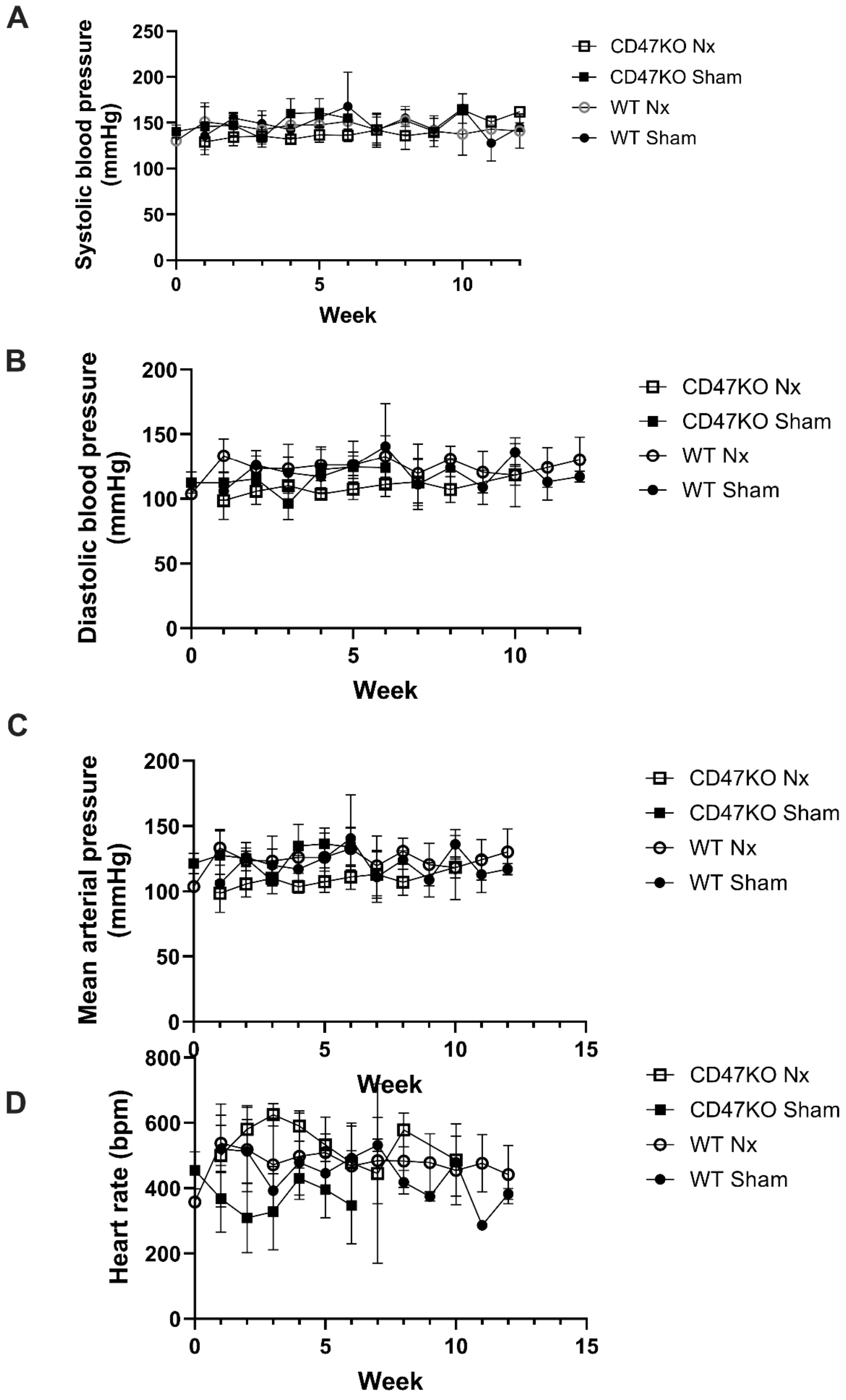

Figure S2. Effects of 5/6-Nx on blood pressure, mean arterial pressure and heart rate.
